# Hepatic Arterial Therapy with Drug-Eluting Beads in the Management of Metastatic Bronchogenic Carcinoma to the Liver: A Multi-Institutional Registry

**DOI:** 10.1155/2012/292131

**Published:** 2012-02-22

**Authors:** Heba Fouad, Tiffany Metzger, Cliff Tatum, Ken Robbins, Robert C. G. Martin

**Affiliations:** ^1^Radiology Department, Kasr Al-Aini Hospital, Faculty of Medicine, Cairo University, Cairo 11950, Egypt; ^2^Division of Surgical Oncology, Department of Surgery, University of Louisville School of Medicine, Louisville, KY 40202, USA; ^3^Norton Radiology, Louisville, KY 40202, USA; ^4^Baptist Health, Little Rock, AR, USA

## Abstract

*Introduction*. There has been limited information reported on the use of hepatic arterial therapy in liver dominant hepatic metastases arising from lung cancer. The aim of this study was to evaluate the safety and efficacy of hepatic arterial therapy in the treatment of liver dominant hepatic metastases arising from lung cancer. *Methods*. Thirteen patients underwent a total of 30 treatment sessions with Drug-Eluting Beads. Eight of the thirteen received only doxorubicin DEB (17 of the total treatments), and four patients received Irinotecan DEB (7 of the total treatments). *Results*. The planned preprocedural dosage was a median of 75 mg (range 19–200), with total hepatic dose exposure being a median of 150 mg (range 0–458), with a technical success rate of 97% in all 29 treatments. There were 4 adverse events related to treatment, but no evidence of hepatic insufficiency. Overall 6-month and 12-month response rates were 50%. After a median followup of 24 months, the median overall survival in this cohort was 14 months (range 7–48 months). *Conclusion*. Drug-eluting beads loaded with doxorubicin (DEBDOX) or irinotecan (DEBIRI) can be safely and effectively used in treatment of patients with liver predominant metastatic disease from lung cancer.

## 1. Introduction

Lung cancer is the leading cause of cancer-related mortality not only in the United States but also around the world [1]. The four major histological types of lung cancer are squamous cell carcinoma (30% to 40% of lung cancers), adenocarcinoma (25% to 30%), nonsmall cell lung carcinoma (less than 10%), and small cell lung carcinoma (15% to 20%). These four types are subdivided into numerous subtypes [2]. Approximately 85% of patients present with squamous or adenocarcinoma defined as nonsmall cell lung cancer (NSCLC) and treatment may consist of surgery, radiation therapy, chemotherapy, or a combination of these modalities depending on tumor stage and the goals of therapy [3]. Distant metastases speak against treatment with curative intent. The most frequent locations of distant metastases are brain, liver, skeleton, lungs, and adrenals [4].

Since at the time of diagnosis 50% of NSCLC patients present with stage IV disease, only palliative treatment can be offered [5]. The success of chemotherapy depends on appropriate selection of the patients. General condition, age, and comorbidities are decisive factors [6]. The combination of platinum with a modern combination partner, which is the optimal chemotherapy, leads to survival times of around 10 months. The chemotherapy not only lengthens life, but in most patients also improves symptoms [7].

Transcatheter arterial embolization (TAE) and chemoembolization (TACE) are increasingly used as regional therapeutic modalities for the treatment of unresectable hepatic malignancies [8]. In general, TAE and TACE have been used when surgical resection and/or systemic therapy have failed to produce an adequate response or when conventional therapy has been known to be ineffective [9]. There has been almost no information reported on the use of TACE in hepatic metastases arising from lung cancer.

Drug-eluting bead TACE is a drug delivery system that combines the local embolization of vasculature with the release of chemotherapy into adjacent tissue. Its administration is similar to that of conventional TACE, and it represents a minimally invasive procedure performed by interventional radiologists [10, 11]. The beads occlude vasculature, causing embolization, and the chemotherapy is delivered locally [12]. Early phase 1 and nonrandomized phase 2 studies have confirmed the ability of this device to deliver a local, controlled, and sustained dose of doxorubicin to the tumors, with minimal systemic doxorubicin exposure [13]. A recently completed randomized Phase 2 study demonstrated that these drug-eluting doxorubicin beads had superior response rates when compared to conventional TACE in advanced HCC and significantly less overall adverse events, including doxorubicin-related side effects [14].

In this study, the aim was to review our experience with drug eluting bead therapy in the multidisciplinary management of patients with liver dominant metastatic lung cancer to the liver.

## 2. Materials and Methods

An IRB-approved prospective multi-institutional treatment registry was reviewed to find patients with lung cancer metastatic to the liver, from which 805 patients undergoing 1358 treatments (TACE) for primary or secondary cancers in the liver were evaluated from January 2007 to Jan 2011. The registry was designed to satisfy the strict criteria for critical appraising of the quality of a registry study with (1) a well-described patient population, (2) hypothesis generating and answering questions, (3) high quality data, with good quality control, (4) independent assessment of outcomes, (5) good clinically relevant followup with minimal loss of patients, and (6) comparable patient evaluation across all institutional participating [15]. Thirteen patients presenting with liver dominant metastatic lung cancer to the liver were treated with doxorubicin or irinotecan drug-eluting beads.

The inclusion criteria were the following patients were included for therapy if they were 18 years of age, of any race or sex, histologic and radiologic (defined as a mass lesion in the liver greater than 1 cm in size) proof of metastatic lung cancer to the liver by percutaneous biopsy, who were able to give informed consent and were eligible for treatment. Patients must have had an ECOG performance status score of less than equal to 2 with a life expectancy of greater than equal to 3 months, nonpregnant with an acceptable contraceptive (defined by the treating physician) in pre-menopausal women. Exclusion to therapy was contraindication to angiographic and selective visceral catheterization, significant extrahepatic disease, representing an eminent life-threatening outcome, greater than 75% of hepatic parenchymal involvement, severe liver dysfunction (defined as presence of ascites or bilirubin >2.5 mg/dL), pregnancy, and severe cardiac comorbidities. Only patients with liver dominant (defined as greater than 50% of the overall total disease burden as measured on baseline pre-DEB treating cross-sectional scanning of the Chest-Abdomen-Pelvis.) were considered for treatment.

Patients were followed for any treatment-related adverse experiences for 30 days after each treatment and monitored for survival for two years. Follow-up assessments included a triphase CT scan of the liver within at least one-to-two months from the treatment completion and then every three months after for the first year and every six months after during the second year, with the evaluation of the enhancement pattern of the target lesion and tumor response rates measured according to modified RECIST criteria [16].

## 3. Image-Guided Infusion Technique

**Figure 1:**
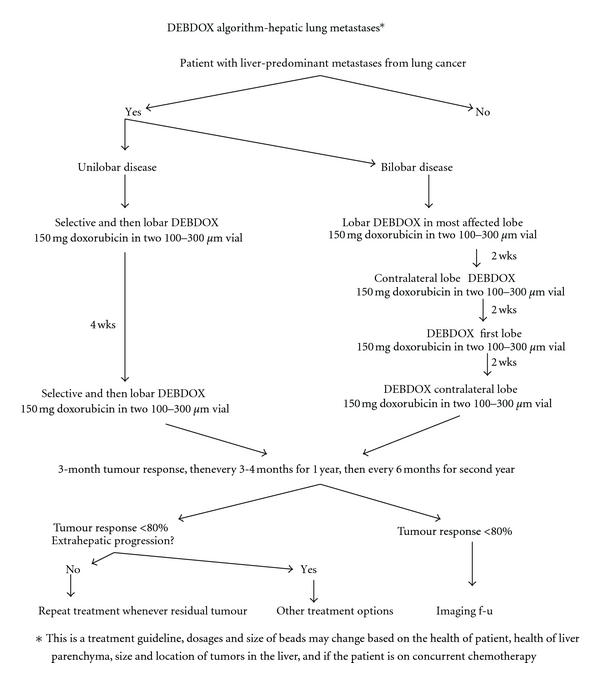
Potential pretreatment DEBDOX treatment algorithm for treating liver dominant metastatic lung cancer. The timing of repeat dosing and the dose utilized may have to be modified based on angiographic findings, patient tolerance, and patient toxicity.

Defining the amount of liver disease was integral to defining both the number of treatments and the type of catheter position and therapy that would be performed. For finite numbers of lesions defined as less than four lesions, a treatment cycle was planned for a minimum of two dosing schedules of at least 100 mg of drug-eluting beads with doxorubicin (DEBDOX) to 150 mg of DEBDOX loaded in two bead vials or 100 mg of drug eluting beads Irinotecan (DEBIRI). Bead sizes of either 100 to 300 microns, 300 to 500 microns, or 500 to 700 microns could be utilized (Figure 1). Treatment intervals were planned for every four to eight weeks. The interval can be extended if causing toxicity to the liver. Based on the extent of liver involvement, two-to-three treatment cycles are planned. A repeat scan is done every three months from the initial first treatment cycle to evaluate response. A treatment cycle is defined as treatment of all liver disease. A treatment is hepatic arterial therapy to one-single lobe, which could also be a treatment cycle if a patient has only unilobar disease. Finite disease was defined as less than 10 total lesions in the liver. The degree of stasis was defined as complete-total loss of arterial flow to the treated segment or lobe, near-complete loss of intratumoral vascularity and near loss of arterial flow to the treated segment or lobe, partial-loss of intratumoral vascularity, and no stasis.

For diffuse disease (bilobar with >25% liver involvement) a plan of a minimum of four-dosing schedule again of 100 to 150 mg (depending on the extent of tumor burden and the extent of hepatic parenchyma reserve) are loaded into two bead vials of the similar size as above. The plan includes at least two treatments per lobe with every three-to-four week dosing schedule. The toxicity effect is followed, and the treatment interval is determined accordingly. Repeat CT scan three months from the first dose to evaluate tumor response. For example, if patients present with bilobar disease, they would receive first bead treatment to right lobe, then three weeks after second bead treatment to left lobe, then three weeks after third bead treatment to right lobe, and then again three weeks later to left lobe. The decision on bead size was up to the treating discretion based on their initial experience with particle size and the degree of stasis that was planned to be delivered at the end of the treatment [17–19]. The reason for lobar infusion is based on the desire for drug delivery and less on inducing stasis in patients with multifocal lobar disease that is not amenable to superselective delivery [20]. Additional embolic material is not usually followed after appropriate treatment but was up to the physician's discretion. Technical success was defined as the ability to deliver at least 75% of the preplanned procedural dose.

All bead therapies were performed with the DC/LC bead microsphere (drug eluting bead (DEB); http://www.biocompatibles.com/, Biocompatibles UK, Surrey, UK). The saline suspension in the DC/LC bead microsphere was removed, and the beads were mixed with doxorubicin solution at a dose of 75 mg per 2 mL at least four hours before the procedure depending on the dose that was planned to be delivered.

### 3.1. Patient and Tumor Characteristics

Thirteen patients underwent a total of 29 treatment sessions with DEBs. Eight of the thirteen received only doxorubicin DEBs (17 of the total treatments), and four patients received Irinotecan DEBs (7 of the total treatments), (Table 1). There were 7 males (54%) and 6 females (46%) in this study, with a median age of 68 years (range 43–72). Past medical histories were significant for prior cardiac disease in three patients, underlying diabetes in three patients (one insulin dependent and two noninsulin dependent), and prior alcohol abuse in two patients as well as tobacco smoking in nine patients. Two patients had vascular disease, seven had pulmonary disease, and eight were hypertensive. Prior surgical histories included prior cholecystectomy in four patients. The Karnofsky performance scale was used to assess the pretreatment health of the patients, and they all ranged (70–100) with a median of 90%.

## 4. Results

The extent of liver involvement was <25% (*n* = 6), 26–50% (*n* = 6), and 51–75% liver involvement in one patient. The median number of target lesions was 4 (range 1–20). The total target lesion size (sum of a maximum of five lesions) was 12.7 cm (range 2.5–21.8 cm).

### 4.1. Treatment

Thirteen patients underwent a total of 29 total bead courses of either DEBDOX or DEBIRI, with the median number of treatments per patient being 2 (range 1–5). The planned preprocedural dosage was a median of 75 mg (range 19–200), with total hepatic dose exposure being a median of 150 mg (range 0–458), with a technical success rate of 97% in all 30 treatments. The most common bead size was 100–300 micron beads in 23 patients (23 vials total), 300–500 micron beads in 5 patients (3.5 vials total), and 500–700 micron beads in 1 patient (1 vial total) (Table 2).

The degree of flow occlusion in the 29 bead courses included no stasis in 5 courses (17%), partial stasis in 7courses (24%), near stasis in 6 courses (21%), and complete stasis in 11 courses (38%).

### 4.2. Patient Tolerance, Morbidity, and Mortality

During the 29 treatments, four adverse events occurred (14%), with one patient developing dehydration and confusion, grades 3 and 2 adverse events, respectively, both of which possibly related to systemic chemotherapy effect. One patient developed angina, a grade 3 adverse event that revolved. Another patient developed hypotension, a grade 2 adverse event that quickly resolved (Table 3).

### 4.3. Follow-Up and Tumor Response

Response rates for all patients were recorded at 3, 6, 9, 12, and 18 months. Response rate was evaluated using modified RECIST. At three months, overall response was 54% with disease control of 70%, with one patient (8%) having a complete response, six patients (46%) having a partial response, stable disease in two patients (15%), and progressive disease in four patients (31%). Location of progression was outside the liver in the lung and bone in separate patients. Two patients died of disease, and one died of other cause at three months. Progression of disease was seen in two patients at 6 months, and three died of disease at 6 months. Progression of disease was seen in the liver in 1 patient with the remaining patients treated having progression to the bone, lymph nodes, or primary tumor. At 9-month followup, the response rate was 20% with disease control of 50% with 2 patients achieving overall response (1 patient CR and 1 patient PD). At the final follow-up time of 18 months, response rate was 40% with one patient with CR, one patient with PR (Table 4). After a median followup of 24 months, the median overall survival in this cohort was 14 months (range 7–48 months).

## 5. Discussion

Liver metastasis from lung cancer is regarded as stage IV disease where only palliative treatment can be offered. Chemotherapy is beneficial for palliation in patients with locally advanced and metastatic disease [21]. There is no reported data concerning transarterial chemotherapy for liver metastasis originating from lung cancer.

Transarterial therapies take advantage of the dual blood supply of the liver. Approximately 80% of the blood supply to hepatic metastases arrives via the hepatic artery, whereas three fourths of the blood supply to normal hepatic parenchyma are portal venous. Hence, cytotoxic agents that are infused selectively into the hepatic artery preferentially target tumor cells over normal hepatic tissue. Transarterial chemoembolization (TACE) is a catheter-based technique that combines both regional chemotherapy and embolization to increase the dwell time of cytotoxic agents and induce ischemia in the tumor. The use of drug-eluting microspheres in a new variation of the TACE method is designed to improve the precision of drug delivery [22]. In our study drug-eluting beads (DC/LC beads) loaded with doxorubicin or irinotecan were used in treatment of liver metastases from lung cancer. Beads are composed of biocompatible polymers such as polyvinyl alcohol (PVA) hydrogel that has been sulphonated to enable the binding of chemotherapy [23]. The beads occlude vasculature, causing embolization, and the chemotherapy is delivered locally [12]. However, drawbacks of this method of treatment include hazards of the procedure itself and those related to the drug-eluting beads such as nausea, vomiting, transient hypertension, pain, liver abscess, and up to mortality.

This treatment so far has been performed in 13 patients, eight of which died of disease progression and one out of complication. The treatment was found to be safe, without any significant adverse events during the treatment phase of the therapy. Systemic progression to lung, bone, and peritoneum was the most common sites where patients failed. The remaining is still in the study, and most of the target lesions are decreasing in size, showing significant improvements on the prognosis of the patients, with no related serious adverse events. These locations of progression demonstrate the need for combination therapy of both systemic therapy and local hepatic arterial therapy given the propensity for even liver-dominant metastatic lung cancer to demonstrate significant progression outside the liver. This unpredictability of progression demonstrates that a monotherapy will have limited overall disease control.

The current literature for systemic chemotherapy to treat liver dominant hepatic metastasis to the liver is limited. The main reason for this is that this is a unique subset of patients that have not been evaluated or recorded in the medical oncology literature. There is a current unmet need of defining how many patients with stage IV lung cancer have liver-dominant disease and thus could possibly derive benefit from local regional hepatic arterial therapy. One comparative study on radiation therapy by Eble et al. evaluated the role of palliative irradiation was in 55 patients with liver metastases from colorectal (*n* = 35), breast (*n* = 10), and lung cancer (*n* = 10). A mean dose of 23.8 Gy was delivered, with daily fractions of 1.5 (*n* = 30), 1.8 (*n* = 1), or 2 Gy (*n* = 16) [24]. Complete and near complete pain relief was obtained in six (28.6%) and nine (42.9%) patients. Median survival was 36.5 days for patients with lung cancer, 70.5 and 73 days for patients with breast and colorectal cancer.

Thus in conclusion, this represents the first ever report of hepatic arterial therapy in the management of liver-dominant metastatic lung cancer. We have demonstrated that drug-eluting beads loaded with Doxorubicin or Irinotecan can be safely and effectively used in treatment of patients with liver-predominant metastatic disease from lung cancer. This should be considered as an alternative or in combination with systemic chemotherapy in selected patients, yielding promising tumor response.

## Figures and Tables

**Table 1:** Clinical characteristics in 13 lung metastatic patients treated with LC beads.

Characteristics	*N* = 13
Age (years) (median, range)	68 (43–72)
Gender	
Male	7 (54%)
Female	6 (46%)
Past medical history	
Cardiac	3 (23%)
Vascular	2 (15%)
Pulmonary	7 (54%)
Diabetes	3 (23%)
Insulin	1 (33%)
NonInsulin	2 (67%)
Alcohol	2 (15%)
Tobacco	9 (69%)
Median packs	60 (40–300)
Hypertension	8 (62%)
Prior cholecystectomy	4 (31%)
Karnofsky performance scale	90% (70–100)
Extent of liver lesions	
Distinct number	11 (85%)
Numerous	2 (15%)
Liver involvement	
<25%	6 (46%)
26–50%	6 (46%)
51–75%	1 (8%)
Number liver tumors (median, range)	4 (1–20)
1	18%
2	9%
≥3	73%
Sum of target lesion(s) size (median, range)	12.7 cm (2.5–21.8)
Lesion location	
Seg 2–4	1 (8%)
Seg 4–8	3 (23%)
Seg 5–8	2 (15%)
Other	7 (54%)

**Table 2:** Bead catheter infusion outcomes.

	*N* = 30 total treatments
Number of bead courses	Median 2 (range 1–5)
Technical success	97%
Dosage delivered (median, range)	75 mg (19–200)
Total hepatic dose exposure	150 (0–458)
1	150 (0–200)
2	150 (79–190)
3	325
>3	458
Bead Size Utilized	
100–300	24
300–500	5
500–700	1
Complications	14%
Extrahepatic infusion	0
Hematologic changes	
WBC	−1.9 (−9994.4–9991.8)
HGB	0.1 (−9989.1–9987.5)
Bilirubin	0 (−9998.7–9998.8)

**Table 3:** Bead infusion-related morbidity.

Side effect (*N* = 4)	All grades	Severe grade*
Confusion	1	—
Dehydration	—	1
Angina	—	1
Hypotension	1	—

*Defined as Grade 3 or higher.

**Table 4:** Response rates* for all 13 patients evaluated.

Response	3 mon *N* = 13	6 mon *N* = 10	9 mon *N* = 8	12 mon *N* = 6	18 mon *N* = 6
Complete response	1	1	1	2	1
Partial response	6	1	1	1	1
Stable disease	2	3	3	3	3
Progression of disease	4	2	1	0	0
Not Reached time point					
DOD	2	3	2	0	1
DOC	1	0	0	0	0

DOD: dead of disease; DOC: dead of complication.

*Response rates measured using modified RECIST criteria.

## References

[1] Molina JR, Yang P, Cassivi SD, Schild SE, Adjei AA (2008). Non-small cell lung cancer: epidemiology, risk factors, treatment, and survivorship. *Mayo Clinic Proceedings*.

[2] Brambilla E, Gazdar A (2009). Pathogenesis of lung cancer signalling pathways: roadmap for therapies. *European Respiratory Journal*.

[3] Schneider BJ (2008). Non-small cell lung cancer staging: proposed revisions to the TNM system. *Cancer Imaging*.

[4] Silvestri GA, Littenberg B, Colice GL (1995). The clinical evaluation for detecting metastatic lung cancer: a meta- analysis. *American Journal of Respiratory and Critical Care Medicine*.

[5] Socinski MA, Crowell R, Hensing TE (2007). Treatment of non-small cell lung cancer, stage IV: ACCP evidence-based clinical practice guidelines (2nd edition). *Chest*.

[6] Hammerschmidt S, Wirtz H (2009). Lung cancer—current diagnosis and treatment. *Deutsches Arzteblatt*.

[7] Socinski MA, Morris DE, Masters GA, Lilenbaum R (2003). Chemotherapeutic management of stage IV non-small cell lung cancer. *Chest*.

[8] Liapi E, Geschwind JFH (2007). Transcatheter and ablative therapeutic approaches for solid malignancies. *Journal of Clinical Oncology*.

[9] Artinyan A, Nelson R, Soriano P (2008). Treatment response to transcatheter arterial embolization and chemoembolization in primary and metastatic tumors of the liver. *HPB*.

[10] Lewis AL, Gonzalez MV, Lloyd AW (2006). DC Bead: in vitro characterization of a drug-delivery device for transarterial chemoembolization. *Journal of Vascular and Interventional Radiology*.

[11] Lewis AL, Taylor RR, Hall B, Gonzalez MV, Willis SL, Stratford PW (2006). Pharmacokinetic and safety study of doxorubicin-eluting beads in a porcine model of hepatic arterial embolization. *Journal of Vascular and Interventional Radiology*.

[12] Taylor RR, Tang Y, Gonzalez MV, Stratford PW, Lewis AL (2007). Irinotecan drug eluting beads for use in chemoembolization: in vitro and in vivo evaluation of drug release properties. *European Journal of Pharmaceutical Sciences*.

[13] Poon RT, Tso WK, Pang RW (2007). A phase I/II trial of chemoembolization for hepatocellular carcinoma using a novel intra-arterial drug-eluting bead. *Clinical Gastroenterology and Hepatology*.

[14] Lammer J, Malagari K, Vogl T (2010). Prospective randomized study of doxorubicin-eluting-bead embolization in the treatment of hepatocellular carcinoma: results of the PRECISION v study. *CardioVascular and Interventional Radiology*.

[15] Levine MN, Julian JA (2008). Registries that show efficacy: good, but not good enough. *Journal of Clinical Oncology*.

[16] Lencioni R, Llovet JM (2010). Modified recist (mRECIST) assessment for hepatocellular carcinoma. *Seminars in Liver Disease*.

[17] Martin RCG, Joshi J, Robbins K (2011). Hepatic intra-arterial injection of drug-eluting bead, irinotecan (DEBIRI) in unresectable colorectal liver metastases refractory to systemic chemotherapy: results of multi-institutional study. *Annals of Surgical Oncology*.

[18] Martin RCG, Joshi J, Robbins K, Tomalty D, O’Hara R, Tatum C (2009). Transarterial chemoembolization of metastatic colorectal carcinoma with drug-eluting beads, irinotecan (DEBIRI): multi-institutional registry. *Journal of Oncology*.

[19] Martin RCG, Howard J, Tomalty D (2010). Toxicity of irinotecan-eluting beads in the treatment of hepatic malignancies: results of a multi-institutional registry. *CardioVascular and Interventional Radiology*.

[20] Brown DB, Gould JE, Gervais DA (2007). Transcatheter therapy for hepatic malignancy: standardization of terminology and reporting criteria. *Journal of Vascular and Interventional Radiology*.

[21] Pfister DG, Johnson DH, Azzoli CG (2004). American society of clinical oncology treatment of unresectable non-small-cell lung cancer guideline: update 2003. *Journal of Clinical Oncology*.

[22] Kalva SP, Thabet A, Wicky S (2008). Recent advances in transarterial therapy of primary and secondary liver malignancies. *Radiographics*.

[23] Tang Y, Taylor RR, Gonzalez MV, Lewis AL, Stratford PW (2006). Evaluation of irinotecan drug-eluting beads: a new drug-device combination product for the chemoembolization of hepatic metastases. *Journal of Controlled Release*.

[24] Eble MJ, Gademann G, Wannenmacher M (1993). The value of radiotherapy for liver metastases. *Strahlentherapie und Onkologie*.

